# Hematological parameters of newborns from diabetic mothers in Gandhi memorial hospital, Addis Ababa, Ethiopia

**DOI:** 10.1371/journal.pone.0324163

**Published:** 2025-05-27

**Authors:** Eden Zinabu, Mikias Negash, Yitagesu Goshu, Tadiyos Kebede, Amanuel Kelem, Aster Tsegaye

**Affiliations:** 1 Department of Medical Laboratory Sciences, College of Health Sciences, Addis Ababa University, Addis Ababa, Ethiopia; 2 Gandhi Memorial Hospital, Addis Ababa, Ethiopia; 3 Department of Medical Laboratory Sciences, Asrat Woldeyes Health Science Campus, Debre Berhan University, Debre Berhan, Ethiopia; University of Diyala College of Medicine, UNITED STATES OF AMERICA

## Abstract

**Background:**

Diabetes mellitus during pregnancy can significantly affect a newborn’s hematological profile. However, the hematological profiles among newborns of diabetic mothers and their clinical and prognostic implications are not well explored. Therefore, this study aimed to determine hematological parameters of newborns from diabetic mothers in Gandhi Memorial Hospital, Addis Ababa, Ethiopia, 2024.

**Methods:**

A comparative cross-sectional study was carried out at Gandhi Memorial Hospital from March to May 2024. A total of 196 newborns -98 from diabetic mothers and 98 from apparently healthy mothers—were conveniently enrolled in the study. A hematological profile test was assayed using a Sysmex XN-550 analyzer from the whole blood of mothers and cord blood of newborns collected in EDTA. Glucose was measured using a Cobas C311 analyzer from serum collected using SST test tube. Data was entered and analyzed using SPSS version 26, with descriptive statistics, proportions, and independent t-tests. A P-value of less than 0.05 was considered statistically significant.

**Results:**

The study found that the mean ± SD value of red blood cell count was 4.87 ± 0.68 vs 3.33 ± 0.98 x10^12^/L (P < 0.001), hemoglobin 17.48 ± 2.69 vs 12.08 ± 3.48 g/dL (P < 0.001), hematocrit 49.83 ± 7.47 vs 36.13 ± 8.97% (P < 0.001), red cell distribution width 18.35 ± 2.68 vs 17.27 ± 1.92% (P < 0.001), platelet distribution width 12.26 ± 1.80 vs 11.69 ± 1.76% (P = 0.028), and platelet large cell ratio was 26.05 ± 5.27 vs 24.07 ± 2.21% (P = 0.001) were significantly higher in newborns born to diabetic mothers compared to controls. Polycythemia was seen in large numbers in newborns born to diabetic mothers (56.1%, 55/98 vs 1%, 1/98). On the other hand, the platelet count and PCT values were significantly lower in newborns from diabetic mothers than controls, with a mean value of 239.69 ± 92.52 (10^3^/µL) and 0.24 ± 0.09 (%), respectively, with a P value < 0.001.

**Conclusion:**

The hematological profile shows a significant difference among newborns from mothers with diabetes and apparently healthy mothers. Therefore, it is better to consider hematological parameters as a screening tool for early detection of hematological complications in newborns from diabetic mothers, and this screening should be encouraged.

## Introduction

Hyperglycemia is a condition linked to a metabolic disorder called diabetes mellitus [1]. Diabetes is a Greek word that describes polyuria, or abundant urination, and mellitus is a Latin word that refers to the sugar found in diabetic patients’ urine [2].

Glucose serves as the energy supply for our blood cells. Under normal circumstances, glucose is transported to the cells by the bloodstream and is subsequently absorbed and utilized by the cells using the hormone insulin, which is created by the pancreatic beta cells. During diabetes mellitus, since there is low insulin or insulin resistance, the glucose in the blood stream will not be absorbed [1].

The symptoms of diabetes mellitus (DM) include polydipsia, polyuria, blurred vision, fatigue, unexplained weight loss, itching, polyphagia, and exhaustion. There are different types of diabetes mellitus [3], Type 1 diabetes mellitus (T1DM) is an autoimmune disorder, Type 2 diabetes mellitus (T2DM) is associated with obesity and old age, and Gestational diabetes mellitus is associated with pregnancy and hormones produced by the placenta [3–5].

Diabetes mellitus has a significant impact on various hematological parameters in diabetic mothers and their newborns. It causes changes in red blood cell (RBC) count, hemoglobin (Hb), hematocrit, red blood cell indices, platelets, and white blood cell count. A study has found decreased RBC count, Hb, and hematocrit levels and increased WBC and platelet counts in diabetic patients compared to healthy controls [6]. Research indicates that newborns from diabetic mothers show increased RBC, Hb, HCT, WBC, red blood cell indices, and a decrease in platelet count. These changes in hematological parameters due to diabetes are thought to be linked to hyperglycemia, inflammation, and oxidative stress associated with the disorder [7].

The mother’s high level of glucose also causes an increase in the glucose level of the fetal environment. When there is hyperglycemia, it triggers several metabolic signaling pathways that lead to inflammation, cytokine secretion, cell death, and oxidative stress. This is caused by increased diacylglycerol (DAG) formation, which activates protein kinase C (PKC) and the NADPH-oxidase system, leading to reactive oxygen species (ROS) production and oxidative stress. In normal conditions, these reactive oxygen species are balanced by the antioxidants that we get from eating vegetables and fruits. But in DM, the amount of reactive oxygen species produced is far greater than that of the antioxidants. Moreover, the activation of the polyol pathway, which competes with glutathione reductase for NADPH, reduces antioxidant capacity, causing alteration in cell membranes, disruption to Hb’s oxygen binding function, and modification to their mechanical properties [8,9].

Hyperglycemia influences the function of phagocytic cells like neutrophils and macrophages. It is well known that bacterial infections cause the release of chemokines, which draw neutrophils and monocytes from the bloodstream to the tissue. This process is referred to as chemotaxis. A variety of molecules are expressed on the endothelial cell surface, which allows the neutrophil and monocytes to be captured and then roll along the endothelium and adhere. The neutrophil and monocytes adhere to the surface and then migrate into the sub-endothelial tissue to the infection site. The neutrophil and macrophage engulf the bacteria and eliminate them via breakdown in the phagosome, a process known as phagocytosis. In a state of hyperglycemia, chemotaxis is reduced. As a result, there is poor adherence. Phagocytosis is also impaired by hyperglycemia, often making diabetic mothers and their babies susceptible to infection [10].

Newborns from diabetic mothers may see an increase in RBC, Hb, and hematocrit. The principal cause of this is that the hyperglycemic environment of the mother affects the environment of the newborn. The hyperglycemic environment causes the synthesis of fetal insulin, which increases the production of RBCs (erythropoiesis) in the fetal liver and bone marrow. The blood might also become more viscous due to the higher concentration of RBCs. Since the number of RBCs is higher, that in turn makes the Hb and hematocrit values higher [11]. This study aimed to determine hematological parameters of newborns from diabetic mothers in Gandhi Memorial Hospital, Addis Ababa, Ethiopia.

## Materials and methods

### Study area, design, and period

A comparative cross-sectional study was conducted among newborns from diabetic mothers from March to May 2024 at Gandhi Memorial Hospital, Addis Ababa, Ethiopia. The hospital is situated in the Kerkos sub-city of central Addis Ababa, Ethiopia. The hospital opened its doors to the public on March 7, 1997. The hospital bears its name after the esteemed Mohandas Karamchand Gandhi. It provides preventive, curative, and diagnostic services to pregnant women and has facilities that measure hematological parameters. The hospital’s diverse prenatal population and large postnatal population made it an ideal location for the study.

### Population

All newborns born from diabetic and apparently healthy mothers who fulfilled the eligibility criteria and attended Gandhi Memorial Hospital during the study period were the study population.

#### Inclusion criteria.

The case group consisted of all newborns from diabetic mothers who attended Gandhi Memorial Hospital during the study period, along with diabetic pregnant mothers who voluntarily participated in the study. In contrast, the control group included all newborns born to apparently healthy mothers who attended the hospital during the same period, as well as apparently healthy pregnant mothers who voluntarily participated.

#### Exclusion criteria.

The case group excluded pregnant mothers with a history of chronic diseases other than diabetes mellitus (such as HIV infection, hypertension, chronic liver disease, chronic kidney disease, chronic heart failure, or malignancy). Additionally, newborns who died shortly after birth or those with complications were also excluded. Similarly, the control group excluded newborns who died shortly after birth and those with complications.

#### Study variables.

Hematological parameters were the dependent variables. Independent variables included socio-demographic variables of the pregnant mother (age, marital status, educational level, monthly income, residence, occupation), duration and type of diabetes mellitus, blood glucose level, blood pressure level, smoking and alcohol habits, physical exercise, Body Mass Index (BMI), number of previous pregnancies, mode of delivery, delivery complications, weight of the newborn, and gestational age.

#### Sample size calculation and sampling method.

The sample size needed was calculated using hypothesis testing for the difference between two population means, resulting in a minimum sample size of 196, which was 98 samples from newborns of diabetic mothers and 98 samples from newborns of apparently healthy mothers. A convenient sampling method was used to enroll volunteer mothers and their newborns.

### Data collection

#### Socio-demographic data.

Participants were asked to give their consent if they were willing to participate in the research after we explained the aim of the study to them. Then, the questionnaire was pretested on 5% of the actual sample size in Zewditu Memorial Hospital to assess the main gaps and to run the study effectively. Next, a nurse who took training from the principal investigator or the principal investigator employed a semi-structured questionnaire to gather clinical and sociodemographic data from research participants who had diabetes and those who did not.

### Sample collection

An eight-milliliter whole blood sample was collected from the mothers into K2-EDTA (dipotassium ethylenediaminetetraacetic acid) and SST (serum separator tube) for hematological profiling and fasting glucose measurements, respectively. Simultaneously, a 3–4 ml cord blood sample was collected from the newborns. Each sample was assigned a unique coded identifier. Hematological analysis was performed using the Sysmex XN-550 at Gandhi Memorial Hospital, which employs flow cytometry, hydrodynamically focused DC detection, and the SLS-Hb method for comprehensive blood cell analysis. Maternal glucose levels were measured using the Cobas C311 system, a clinical chemistry analyzer that utilizes a UV test at 340 nm to detect glucose in serum and plasma.

### Data quality assurance

To ensure data reliability and validity, a multi-faceted approach was implemented throughout the study. The questionnaire, initially drafted in English, was translated into Amharic and back-translated to English to confirm accuracy and cultural relevance. Before data collection, the questionnaire was pretested with 5% of the sample at Zewditu Memorial Hospital, leading to minor revisions for clarity. Trained clinical nurses, supervised by the investigator, collected sociodemographic and clinical data, while medical laboratory technologists performed laboratory tests. Throughout the data collection process, the investigator closely monitored procedures, providing ongoing feedback to data collectors to maintain completeness, accuracy, and consistency.

Stringent quality assurance measures were implemented across all phases of laboratory analysis. During the pre-analytical phase, adherence to standard operating procedures (SOPs) was ensured during sample collection, with the principal investigator supervising the process. Samples were inspected for clots, hemolysis, and proper volume. Instrument status, reagent integrity, and environmental conditions were checked regularly. In the analytical phase, three levels of hematological cell controls were run before analyzing participant samples, and SOPs were strictly followed. Post-analytical quality was maintained by using the analyzer’s printed results to minimize clerical errors. All data were double-checked, recorded as exact values, and interpreted using company-provided reference intervals.

### Data analysis and interpretation

Following data collection and completeness checks, the data was entered into EpiData and subsequently transferred to SPSS version 26 for analysis. The Shapiro-Wilk test was employed to assess the normal distribution of the data. Descriptive statistics were used to summarize socio-demographic characteristics, while proportions were used to report the magnitude of hematological abnormalities. Independent t-tests were conducted to compare hematological and clinical profiles between the case and control groups. Statistical significance was determined using a P-value threshold of less than 0.05 to identify associations between diabetes mellitus and hematological changes.

### Operational definitions

- **Body mass index:** It is measured by the ratio of weight to height^2^. The measurement used a well-standardized and calibrated balance. The height measurement was calibrated and had a good standard meter measurement. BMI was calculated by the WHO-recommended formula: BMI = weight/height^2^, with a reference range of 18.5–24.9 kg/m^2^ for pregnant mothers.
- **Blood pressure:** Blood pressure was measured by an instrument called a sphygmomanometer cuff. The cuff was well standardized, i.e., it fulfilled the AAMI/ESH/ISO standard protocol. Normotensive mother Is a Systolic Blood Pressure (SBP) between 90 and 120 mmHg and a Diastolic Blood Pressure (DBP) between 60 and 80 mmHg.
- **Healthy (non- diabetic) mother:** The expected value for normal fasting blood glucose concentration is between 70 and 100 mg/dl.
- **Diabetic mother:** The expected values for normal fasting blood glucose concentration are > 126 mg/dl.

#### For mothers.

- **Leukocytosis:** the number of WBCs (leukocyte count) >15*10^3^/ul
- **Leukocytopenia**: the number of WBCs (leukocyte count) <3*10^3^/ul
- **Anemia:** A Hb level of <11.7 g/dl in the blood
- **Polycythemia:** A hematocrit level of >48%
- **Thrombocytopenia:** Platelet count of <150*10^3^/ul
- **Thrombocytosis:** A condition in which platelets count >440*10^3^/ul

#### For newborns.

- **Leukocytosis:** the number of WBCs (leukocyte count) >30*10^3^/ul
- **Leukocytopenia:** the number of WBCs (leukocyte count) <9*10^3^/ul
- **Anemia:** A Hb level of <13.4 g/dl in the blood
- **Polycythemia:** A hematocrit level of >65%
- **Thrombocytopenia:** Platelet count of <150*10^3^/ul
- **Thrombocytosis:** A condition in which platelets count >440 *10^3^/ul

### Ethical considerations

The study was conducted after receiving ethical approval from the Department of Medical Laboratory Sciences, College of Health Sciences, Addis Ababa University, and permission was obtained from Gandhi Memorial Hospital. The study was conducted in accordance with the Declaration of Helsinki. All participants were informed about the purpose, procedures, potential risks, and benefits of the research. Informed consent was obtained from all participants prior to the collection of clinical data and blood samples. Consent was obtained in written form; participants signed a consent form before enrollment. For the newborns, written consent was obtained from their parents or legal guardians. Participation was entirely voluntary, and individuals were informed they could withdraw from the study at any time without penalty. Confidentiality was ensured by linking participant data to study code numbers, and only authorized study personnel had access to the data. Any abnormal test results were communicated to the participants’ attending physicians.

## Result

### Socio-demographic and clinical characteristics

In this study, a total of 196 newborns (98 from diabetic mothers and 98 from apparently healthy controls) were involved. The majority of the diabetic and healthy mothers were between 26 and 30 years old (51% and 40.8%, respectively). Almost all the diabetic and non-diabetic mothers were urban dwellers. Of the 98 diabetic mothers, 45 (45.9%) were housewives, and of the 98 healthy mothers, 64 (65.3%) of the healthy controls were housewives; the majority 42 (42.9%) of cases and 37 (37.8%) of control mothers attained secondary school (**Table 1**).

**Table 1:** Socio-demographic characteristics of the newborns from diabetic and healthy mothers at Gandhi Memorial Hospital from March to May 2024, Addis Ababa, Ethiopia.

Variables	Categories	Case group N(%)	Control group N(%)
Age of the mothers (Years)	Under 20	3 (3.1)	7 (7.1)
	20-25	21 (21.4)	33 (33.7)
	26-30	50 (51.0)	40 (40.8)
	31-35	18 (18.4)	12 (12.2)
	36 and above	6 (6.1)	6 (6.1)
Residence of the mothers	Urban	98 (100)	96 (98.0)
	Rural	0 (0)	2 (2.0)
Occupation of the mothers	House wife	45 (45.9)	64 (65.3)
	Government employee	34 (34.7)	19 (19.4)
	Private business owner	19 (19.4)	14 (14.3)
	Student	0(0)	1 (1.0)
Marital status of the mothers	Married	89 (90.8)	98 (100)
	Divorced	2 (2.0)	0 (0)
	Single	7 (7.1)	0 (0)
Education Background	Can’t read or write	5 (5.1)	12 (12.2)
	Primary	28 (28.6)	33 (33.7)
	Secondary	42 (42.9)	37 (37.8)
	Diploma and above	23 (23.5)	16 (16.3)
Monthly family income	<1500 Birr	0 (0)	1 (1.0)
	1501-5000 Birr	39 (39.8)	45 (45.9)
	5001-10000 Birr	53 (54.1)	52 (53.1)
	>10000 Birr	6 (6.1)	0 (0)

### Clinical characteristics of study participants

Around 23.5% of diabetics and 36.7% of healthy mothers had taken iron, folate, or vitamin supplements. There was no history of drinking alcohol or smoking cigarettes in healthy mothers, while alcohol and cigarettes were consumed by 2% and 1% of diabetic mothers, respectively. About 57.1% of healthy mothers had delivered their babies via cesarean section, compared to 60.2% of diabetic mothers. Complication during delivery was around 17.3% in cases and around 15.3% in controls. The mean BMI of diabetic mothers was 29.32 ± 2.87 kg/m^2^, while that of healthy mothers was 27.92 ± 2.72 kg/m^2^. The mean SBP of diabetic mothers was 111.64 ± 6.94 mmHg, while that of healthy mothers was 111.57 ± 7.72 mmHg. The mean DBP of diabetic mothers was 73.12 ± 6.31 mmHg, while it was 72.81 ± 5.97 mmHg in healthy mothers. The mean gestational ages of delivery in the cases were 39.68 ± 1.23 weeks and 37.48 ± 2.17 weeks in controls. The mean birth weights of babies in cases were 3.68 ± 0.36 kilograms, while in controls, they were 3.05 ± 0.49 kilograms (**Table 2**).

**Table 2:** The clinical characteristics of the newborns from diabetic and healthy mothers at Gandhi Memorial Hospital from March to May 2024, Addis Ababa, Ethiopia.

Variables	Category	Case group N (%)	Control group N (%)
Exercise	Yes	13 (13.3)	15 (15.3)
	No	85 (86.7)	83 (84.7)
Hourly exercise per week	1-4 hours	13 (13.3)	6 (40.0)
	4-8 hours	0 (0)	7 (46.7)
	8-10 hours	0 (0)	1 (6.7)
	>10 hours	0 (0)	1 (6.7)
Alcohol consumption	Yes	2 (2.0)	0 (0)
	No	96 (98)	98 (100)
Alcohol consumption per week	Once a week	1 (1.0)	0 (0)
	Twice a week	1 (1.0)	0 (0)
Smoking habit	Yes	1 (1.0)	0 (0)
	No	97 (99.0)	98 (100)
SBP of mothers in mmHg^*^		111.64 ± 6.94	111.57 ± 7.72
DBP of mothers mmHg^*^		73.12 ± 6.31	72.81 ± 5.97
Glucose level^*^		163.82 ± 32.22	90.21 ± 8.71
BMI of mothers in kg/m^2^^*^		29.32 ± 2.87	27.92 ± 2.72
Take supplements (Vitamin D, Vitamin B12, folate, Iron)	Yes	23 (23.5)	36 (36.7)
	No	75 (76.5)	62 (63.3)
Duration of supplement intake	2 months	1(4.3)	4 (11.1)
	3 months	6 (26.1)	19 (52.8)
	4 months	10 (43.5)	5 (13.9)
	5 months	3 (13)	7(19.4)
	6 months	3 (13)	1 (2.8)
Total number of pregnancies	1	35 (35.7)	41 (41.8)
	2	46 (46.9)	30 (30.6)
	3	14 (14.3)	20 (20.4)
	4	3 (3.1)	5 (5.1)
	More than 4	0 (0)	2 (2.0)
Gestational age of delivery in weeks^*^		39.68 ± 1.23	37.48 ± 2.17
Mode of delivery	Vaginal birth	39 (39.8)	42 (42.9)
	CS birth	59 (60.2)	56 (57.1)
Weight of newborn in kilograms^*^		3.68 ± 0.36	3.05 ± 0.49

SBP: Systolic blood pressure, DBP: Diastolic blood pressure, BMI: Body mass index NB:

* indicates the result presented by mean ± SD

In this study, 69.4% of the diabetic mothers had gestational diabetes, while 30.6% of the diabetic mothers had type II diabetes. Of the diabetic mothers, 45.9% of them take metformin, while the remaining 54.1% use no medication for their diabetes (**Table 3**).

**Table 3:** The clinical characteristics of diabetic mothers at Gandhi Memorial Hospital from March to May 2024, Addis Ababa, Ethiopia.

Variable	Category	Case N (%)
Type of diabetes mellitus	Type I diabetes	0 (0)
	Type II diabetes	30 (30.6)
	Gestational diabetes	68 (69.4)
Duration of diabetes mellitus	2 months-5 months	69 (70.4)
	1 year-2 and half years	8 (8.2)
	3 years-6 years	21 (21.4)
Medication for diabetes mellitus	Metformin	45 (45.9)
	Nothing	53 (54.1)

### Hematological profiles of mothers

The mean platelet count of diabetic mothers was 235.21 ± 111.21 (10^9^/L), while that of healthy mothers was 207.62 ± 77.80 (10^9^/L). The mean P-LCR of diabetic and healthy mothers was 29.85 ± 8.10 (%) and 24.87 ± 2.44 (%), respectively. The immature granulocytes (IG count and %) were 0.10 ± 0.17 x10^9^/L versus 0.05 ± 0.06 x10^9^/L and 0.76 ± 0.92% versus 0.48 ± 0.46% for cases and controls, respectively. The platelet (P = 0.046), IG (P = 0.009), and P-LCR (P < 0.001) were significantly higher in diabetic mothers than in healthy mothers (**Table 4**).

**Table 4:** Hematological profile of healthy and diabetic mothers attending Gandhi Memorial Hospital from March to May 2024, Addis Ababa, Ethiopia.

Hematological parameters	Diabetic mothers (n = 98) Mean ± SD	Healthy mothers (n = 98) Mean ± SD	P-value
WBC (10^9^/L)	11.24 ± 4.25	11.59 ± 5.07	0.603
RBC (10^12^/L)	3.95 ± 0.62	3.92 ± 0.56	0.691
Hb (g/dL)	12.22 ± 1.80	12.19 ± 1.75	0.888
Hematocrit (%)	35.42 ± 5.04	35.17 ± 5.00	0.732
MCV (fL)	89.11 ± 7.47	89.73 ± 6.02	0.522
MCH (pg)	31.07 ± 2.52	31.06 ± 2.53	0.991
MCHC (g/dl)	34.68 ± 1.42	34.55 ± 1.03	0.489
Platelet (10^9^/L)	235.21 ± 111.21	207.62 ± 77.80	0.046*
RDW-CV (%)	13.67 ± 1.68	13.67 ± 1.75	0.997
PDW (fL)	12.54 ± 1.22	12.93 ± 2.60	0.183
MPV (fL)	10.85 ± 1.05	10.84 ± 1.14	0.928
P-LCR (%)	29.85 ± 8.10	24.87 ± 2.44	<0.001*
PCT (%)	0.24 ± 0.10	0.22 ± 0.07	0.054
NEUT (10^9^/L)	8.75 ± 4.08	9.04 ± 4.91	0.657
LYMPH (10^9^/L)	1.70 ± 0.71	1.69 ± 0.66	0.960
MONO (10^9^/L)	0.66 ± 0.29	0.72 ± 0.38	0.248
EOS (10^9^/L)	0.08 ± 0.14	0.11 ± 0.19	0.278
BASO(10^9^/L)	0.02 ± 0.02	0.02 ± 0.01	1.000
IG (#) (10^9^/L)	0.10 ± 0.17	0.05 ± 0.06	0.009*
IG (%)	0.76 ± 0.92	0.48 ± 0.46	0.009*

**Note:** WBC: White blood cells, RBC: Red blood cells, Hb: Hemoglobin, MCV: Mean cell volume, MCH: Mean cell hemoglobin, MCHC: Mean cell hemoglobin concentration, RDW: Red cell distribution width, PDW: Platelet distribution width, MPV: Mean platelet volume, P-LCR: Platelet-large cell ratio, PCT: plateletcrit, NEUT: Neutrophil, LYMPH: Lymphocyte, MONO: Monocyte, EO: Eosinophil, BASO: Basophil, IG: Immature granulocyte, fL: femtoliter. NB: * indicates statistically significant at p-value <0.05, derived from two sided independent test.

### Hematological profiles of newborns

The mean RBC count of cases was 4.87 ± 0.68 (10^12^/L), while in the control group, it was 3.33 ± 0.98 (10^12^/L). The mean Hg of cases was 17.48 ± 2.69 (g/dL), while that of controls was 12.08 ± 3.48 (g/dL). The RBC (P < 0.001), Hg (P < 0.001), hematocrit (P < 0.001), RDW-SD (P = 0.049), RDW-CV (P = 0.001), PDW (P = 0.028), and P-LCR (P = 0.001) were significantly higher in cases than controls. On the other hand, platelets 239.69 ± 92.52 versus 384 ± 126.97, P < 0.001) and PCT 0.24 ± 0.09 versus 0.39 ± 0.14 (P < 0.001) were significantly lower in cases compared to controls (**Table 5**).

**Table 5:** Hematological profile of newborns from diabetic and healthy mothers attending at the Gandhi Memorial Hospital from March to May 2024, Addis Ababa, Ethiopia.

Hematological parameters	Diabetic group (n = 96) Mean ± SD	Control group (n = 96) Mean ± SD	P-value
WBC (10^9^/L)	14.26 ± 5.95	14.65 ± 6.87	0.672
RBC (10^12^/L)	4.87 ± 0.68	3.33 ± 0.98	**<0.001** ^*^
Hb (g/dL)	17.48 ± 2.69	12.08 ± 3.48	**<0.001** ^*^
Hematocrit (%)	49.83 ± 7.47	36.13 ± 8.97	**<0.001** ^*^
MCV (fL)	102.59 ± 6.70	101.51 ± 9.94	0.374
MCH (pg)	36.54 ± 3.90	35.94 ± 2.38	0.192
MCHC (g/dL)	35.77 ± 1.52	35.07 ± 0.83	**<0.001** ^*^
Platelet (10^9^/L)	239.69 ± 92.52	384 ± 126.97	**<0.001** ^*^
RDW-SD (fL)	62.87 ± 8.35	60.46 ± 8.67	**0.049** ^*^
RDW-CV (%)	18.35 ± 2.68	17.27 ± 1.92	**0.001** ^*^
PDW (fL)	12.26 ± 1.80	11.69 ± 1.76	**0.028** ^*^
MPV (fL)	10.47 ± 0.96	10.28 ± 0.82	0.141
P-LCR (%)	26.05 ± 5.27	24.07 ± 2.21	**0.001** ^*^
PCT (%)	0.24 ± 0.09	0.39 ± 0.14	**<0.001** ^*^
NEUT (#) (10^9^/L)	8.41 ± 5.25	8.47 ± 5.88	0.946
LYMPH (#) (10^9^/L)	4.31 ± 1.64	4.01 ± 2.24	0.288
MONO (#) (10^9^/L)	1.42 ± 0.65	1.51 ± 0.63	0.325
EO (#) (10^9^/L)	0.28 ± 0.25	0.26 ± 0.26	0.744
BASO (#) (10^9^/L)	0.09 ± 0.22	0.08 ± 0.07	0.444
NEUT (%)	52.08 ± 17.67	55.09 ± 17.33	0.231
LYMPH (%)	33.40 ± 13.50	30.93 ± 13.89	0.208
MONO (%)	10.73 ± 4.66	11.65 ± 5.36	0.202
EO (%)	2.48 ± 2.45	2.27 ± 2.28	0.534
BASO (%)	0.71 ± 1.42	0.56 ± 0.42	0.339
IG (#) (10^9^/L)	0.28 ± 0.42	0.17 ± 0.31	0.058
IG (%)	1.39 ± 1.72	1.06 ± 1.51	0.147

**Note:** WBC: White blood cells, RBC: Red blood cells, Hb: Hemoglobin, MCV: Mean cell volume, MCH: Mean cell hemoglobin, MCHC: Mean cell hemoglobin concentration, RDW: Red cell distribution width, PDW: Platelet distribution width, MPV: Mean platelet volume, P-LCR: Platelet-large cell ratio, PCT: plateletcrit, NEUT: Neutrophil, LYMPH: Lymphocyte, MONO: Monocyte, EO: Eosinophil, BASO: Basophil, IG: Immature granulocyte, fL: femtoliter.

* indicates statistically significant at p-value <0.05, derived from two-sided independent t-test

## Discussion

Diabetes mellitus during pregnancy can significantly affect a newborn’s hematological profile. In the current study, babies from mothers with diabetes had mean RBC and Hb values that were greater than those of babies from healthy mothers. Studies carried out in the USA, Japan, Serbia, Bangladesh, and India revealed similar results [12,13,14,15,16,17]. This is most likely due to the possibility of high glucose levels in a fetus whose mother has diabetes, especially if the condition is poorly managed [18]. The increased synthesis of fetal insulin due to this excess glucose drives the production of RBCs (erythropoiesis) in the fetal liver and bone marrow. The increased concentration of RBCs may also cause the blood to thicken. This increased viscosity may affect the circulation and oxygen transfer to tissues, thus raising Hb [19].

In this study, the mean HCT of newborns from diabetic mothers was higher than that of newborns from apparently healthy mothers. Polycythemia was highly prevalent in newborns from diabetic mothers (55/98 versus 1/98 for newborns from diabetic and healthy mothers, respectively). This was in agreement with studies done in Mexico, the USA, Norway, Poland, Hungary, Bangladesh, Turkey, and India [20,21,22,23,24,25,26,27]. When an infant is born to a diabetic mother, the fetus also experiences a rise in glucose. Fetal insulin rises to make up for the increase in glucose.

Then, RBC production in the fetal liver and bone marrow is initiated by insulin. Additionally, this raises the hematocrit level. Other factors contributing to the rise in HCT include oxygen deprivation or fetal hypoxia caused by impaired placenta function or problems with the mother’s vessels as a result of diabetes mellitus [19]. Fetal hypoxia causes the release of erythropoietin, a hormone that stimulates the production of RBCs, in an attempt to compensate for what it believes to be a lack of oxygen. This results in the development of polycythemia [28].

The current study showed that the mean MCHC of newborns from diabetic mothers was higher than that of newborns from healthy mothers. Research done in India [17] recorded a similar result. Since an increase in the production of RBC and Hb occurs in newborns from diabetic mothers, the MCHC also shows an increase in Hb [16].

The current study also revealed that the mean RDW-SD and RDW-CV of newborns from diabetic mothers were higher than those from healthy mothers. This study finding is similar to studies conducted in the USA, Bangladesh, and India [16,17,29]. The probable reason for this could be placental-mediated hemolysis, gestational anemia, and diabetic-related oxidative stress; hence, to compensate for this, there would be enhanced erythropoiesis and premature release of RBCs into the circulation, which would manifest as higher RDW-SD and RDW-CV [30]. Since maternal diabetes has an effect on the fetal environment, fetal hypoxia occurs due to complications of maternal diabetes mellitus, like impaired placental function or maternal vascular complications. The hypoxia triggers the release of erythropoietin, and RBC production starts. These maternal factors might impact the development and maturation of RBC, contributing to a high RDW, which is a marker of anisocytosis [17].

In the present study, the mean platelet count and PCT of newborns from diabetic mothers were lower compared to newborns from apparently healthy mothers. This study’s findings were similar to those of studies conducted in the USA and India [12,31]. Diabetes complications could likely lead to placental insufficiency and platelet consumption, leading to thrombocytopenia. The PLC-R is significantly higher in the current study in newborns of diabetic mothers, which implies that premature platelets are being released to compensate for the consumed ones. Most newborns from diabetic mothers have a decreased platelet count [12], but in some cases, thrombocytosis might occur as a compensatory response to the increased production of RBC in the bone marrow. Other studies done in Serbia [15] recorded that those newborns from diabetic mothers had a higher platelet count. Maternal diabetes might also increase the risk of other complications during pregnancy, so in response to these conditions, the body will produce platelets. Medications that manage diabetes mellitus could also, in turn, increase the production of platelets [32].

In this study, the mean PDW and P-LCR of newborns from diabetic mothers were higher compared to newborns from apparently healthy mothers. This study finding is similar to studies conducted in the USA and India [12,31]. Alterations in platelet parameters have been reported in infants born to diabetic mothers in some studies. These alterations include changes in platelet size distribution (reflected by PDW) and the proportion of large platelets (reflected by P-LCR). In summary, the available evidence suggests that IDMs likely have higher P-LCR and PDW compared to controls as a result of the decreased platelet counts and increased immature platelet production that occur in these infants [33].

In the current study, diabetic mothers had significantly higher platelet counts than non- diabetic mothers. Studies carried out in Saudi Arabia, Nigeria, and Ethiopia showed similar results [34,35,36]. The probable reason for the increase in platelet count in diabetic mothers could be the non-enzymatic glycation of proteins on the surface of platelets by hyperglycemia. This decreases membrane fluidity and causes platelets to become activated. When platelets are activated, they aggregate, and they are removed by the spleen. To compensate for this consumption, platelet production increases. Another reason for the increase in platelet count in diabetic mothers could be platelet activation and consumption induced by increased inflammation. Inflammation is very common in diabetic people, as hyperglycemia and insulin resistance lead to the activation of inflammatory signaling pathways and the release of pro-inflammatory cytokines. Then, inflammation stimulates the bone marrow to produce more platelets to compensate for the increased platelet consumption due to inflammation. This decreases membrane fluidity and causes platelets to become activated. When platelets are activated, they aggregate, and they are removed by the spleen. To compensate for this consumption, platelet production increases. Another reason for the increase in platelet count in diabetic mothers could be platelet activation and consumption induced by increased inflammation. Inflammation is very common in diabetic people, as hyperglycemia and insulin resistance lead to the activation of inflammatory signaling pathways and the release of pro-inflammatory cytokines. Then, inflammation stimulates the bone marrow to produce more platelets to compensate for the increased platelet consumption due to inflammation [37–40]. On the other hand, studies done in India [41,42] showed a decrease in platelet count in diabetic mothers compared to apparently healthy mothers. This can be best explained by the fact that diabetes is associated with increased platelet activation and aggregation, leading to higher platelet consumption and a decrease in platelet count. Hyperglycemia and other metabolic disturbances in diabetes can impair the bone marrow’s ability to produce new platelets, resulting in lower platelet counts. Diabetes can also lead to increased platelet destruction through various mechanisms, such as immune-mediated platelet clearance or increased platelet apoptosis. Diabetic microvascular complications, like nephropathy and retinopathy, can contribute to platelet consumption and decreased platelet counts [41,43].

In this study, the immature granulocyte count of diabetic mothers was significantly higher than that of apparently healthy mothers. This finding was similar to studies done in India and Ethiopia [36,44,45]. Diabetic people are susceptible to inflammation since hyperglycemia and insulin resistance activate inflammatory signaling pathways and the release of pro-inflammatory cytokines. The fact that diabetic people are prone to inflammation sets off the bone marrow to produce more granulocytes to fight off the cause of the inflammation. But since the granulocytes might not have enough time to mature, immature granulocytes are detected in diabetic mothers than apparently healthy mothers [40,43,44].

In the present study, the P-LCR of diabetic mothers was significantly higher than that of apparently healthy mothers. This finding was similar to studies done in Saudi Arabia, Nigeria, and India [34,35,42]. Through several processes, including non-enzymatic glycation of platelet proteins and stimulation of platelet signaling pathways, chronic hyperglycemia in diabetes causes enhanced platelet activation and hyperreactivity. The discharge of bigger, more reactive platelets as a result of this increased platelet activation raises the P-LCR. Moreover, Chronic inflammation and elevated oxidative stress are two features of diabetes that can encourage platelet activation and the generation of bigger, more reactive platelets [37–40].

## Conclusion

The RBC, Hg, hematocrit, MCHC, RDW-CV, PDW, and P-LCR were significantly higher in cases than controls. On the other hand, platelets and PCT were significantly lower in newborns from diabetic mothers than newborns from apparently healthy mothers. The prevalence of polycythemia and thrombocytopenia were higher in newborns from diabetic mothers than in newborns from healthy mothers.

The platelet count, P-LCR, and immature granulocytes were significantly higher in diabetic mothers than apparently healthy mothers. The prevalence of leukocytosis, Anemia, and thrombocytosis were higher in diabetic mothers than apparently healthy mothers.

## Supporting information

S1 Data Excel raw data file. (XLSX)

## Abbreviations

CBC: Complete Blood Count
WBC: White blood cells
RBC: Red blood cells
Hb: Hemoglobin
MCV: Mean cell volume
MCH: Mean cell hemoglobin
MCHC: Mean cell hemoglobin concentration
RDW: Red cell distribution width
PDW: Platelet distribution width
MPV: Mean platelet volume
P-LCR: Platelet-large cell ratio
PCT: Plateletcrit
NEUT: Neutrophil
LYMPH: Lymphocyte
MONO: Monocyte
EO: Eosinophil
BASO: Basophil
IG: Immature granulocyte.
